# Defining and validating regenerative farm systems using a composite of ranked agricultural practices

**DOI:** 10.12688/f1000research.28450.1

**Published:** 2021-02-15

**Authors:** Tommy L.D. Fenster, Claire E. LaCanne, Jacob R. Pecenka, Ryan B. Schmid, Michael M. Bredeson, Katya M. Busenitz, Alex M. Michels, Kelton D. Welch, Jonathan G. Lundgren

**Affiliations:** 1 Blue Dasher Farm, Ecdysis Foundation, Estelline, South Dakota, 57234, USA; 2 California State University East Bay, Hayward, California, USA; 3 Center for Agriculture, Food, and Natural Resources, University of Minnesota Extension, Minneapolis, Minnesota, USA; 4 Department of Entomology, Purdue University, West Lafayette, Indiana, USA; 5 Department of Entomology, University of Nebraska, Lincoln, Nebraska, USA; 6 Department of Natural Resource Management, South Dakota State University, Brookings, South Dakota, 57007, USA

**Keywords:** Biodiversity, Haney Test, Invertebrates, Regenerative Agriculture Label, Soil Carbon, Soil Health, Soil Microbiology

## Abstract

**Background:** Ongoing efforts attempt to define farms as regenerative to aid marketers, policymakers, farmers, etc. The approach needs to balance precision with function, and must be transparent, simple, scalable, transferable, incorruptible, and replicable.

**Methods:** We developed practice-based scoring systems to distinguish regenerative cropland and rangeland, and validate them based on whether these scores scaled with regenerative goals on actual farm operations. Study systems included cornfields of the Upper Midwest, almond orchards of California, and rangeland systems of the Northern Plains. Response variables included soil carbon and organic matter, soil micronutrients, water infiltration rates, soil microbial communities, plant community structure, invertebrate community structure, pest populations, yields, and profit.

**Results:** Regenerative outcomes were strongly correlated with our approach to farm scoring. Soil organic matter, fine particulate organic matter, total soil carbon, total soil nitrogen, phosphorous, calcium and sulfur all increased alongside regenerative matrix scores in one or both of the cropping systems. Water infiltration rates were significantly faster in more regenerative almond orchards. Soil bacterial biomass and Haney soil health test scores were higher as cropland incorporated more regenerative practices. Plant species diversity and biomass increased significantly with the number of regenerative practices employed on almonds and rangelands. Invertebrate species diversity and richness were positively associated with regenerative practices in corn, almonds, and rangelands, whereas pest populations and almond yields were unaffected by the number of regenerative practices. Corn yields were negatively associated with more regenerative practices, while almond yields were unaffected by the number of regenerative practices. Profit was significantly higher on more regenerative corn and almond operations.

**Conclusions:** Our scoring system scaled positively with desired regenerative outcomes, and provides the basis for predicting ecosystem responses with minimal information about the farming operation. Natural clusters in the number of regenerative practices used can be used to distinguish regenerative and conventional operations.

## Introduction

The term “regenerative agriculture” was first coined by Robert Rodale (1983). The name highlighted how industrialized agriculture was severely reducing its natural resource base, and that without rebuilding that natural resource base, “sustainable agriculture” and “conservation agriculture” were insufficient for supporting the food and natural resource needs of a growing human population. Regenerative agricultural systems increase soil health and promote biodiversity while producing nutritious food profitably. Some outcomes of a regenerative operation are improved greenhouse gas relationships, balanced water relationships, reduced pollution from agrichemicals, increased resiliency of farms, more nutritionally robust foods, increased ecosystem services, etc. ( Elevitch *et al.*, 2018; Francis *et al.*, 1986; Sherwood & Uphoff, 2000). A central challenge for researchers, policy makers, consumers, etc. is defining what a regenerative farm is and having a standardized method for discerning them from conventional farms ( Newton *et al.*, 2020; Schreefel *et al.*, 2020).

The principles and philosophy that define regenerative systems have been well formulated by practitioners and educators ( Lal, 2020; Schreefel *et al.*, 2020; UnderstandingAG, 2020; USDA-NRCS, 2020). Essentially, regenerative practices can be distilled down to two central principles: 1) reduce uniform disturbance (such as tillage and agrichemical use), and 2) increase diversity (biodiversity, and the diversity of revenue streams from an operation). The first working formula for these principles was proposed by LaCanne & Lundgren (2018) and included four principles: 1) reduce or eliminate tillage, 2) never leave bare soil, 3) maximize plant diversity and productivity on a farm, and 4) integrate livestock and cropping operations. Here, we add a fifth principle: 5) reduce or eliminate synthetic agrichemicals. In croplands, practices such as no-till, diverse crop rotations lasting more than four seasons, cover cropping, intercropping and interseeding, and livestock integration all contribute to a regenerative farming model ( LaCanne & Lundgren, 2018; Rhodes, 2017). In rangelands, practices that actualize regenerative principles include reduction/elimination of synthetic agrichemicals and adaptive multi-paddock grazing systems that allow a rangeland to rest following the punctuated disturbance of grazing ( Teague & Kreuter, 2020; Wagner *et al.*, 2020). Regenerative farms are also diverse and complementary in their enterprises, and adaptive in their management choices, ensuring that a farm is resilient and profitable in the face of adversity. These practices are dependent upon one another within a system for them to be optimally successful, and it is the system, not the individual practices, that drives the success of an operation.

Classifying regenerative farming operations has to balance precision with function. Adaptability and innovation are hallmarks of operations that are guided by regenerative principles. Therefore, while a matrix of responses or practices must have sufficient nuance to accurately reflect a regenerative system, it cannot be a rigid formula that inhibits that critical adaptability and ongoing innovation. Nor should a scoring system be so complex as to exclude adoption by regenerative farmers. Classification schema have been developed for other movements within ecological agriculture, including conservation agriculture ( Hendrix *et al.*, 1986; Kassam *et al.*, 2012), organic agriculture ( USDA-AMS, 2020), holistic management ( Alfaro-Arguello *et al.*, 2010), mob grazing ( Gurda *et al.*, 2018), etc. In these systems, the tangible manifestations of core principles are represented by a series of standards or practices. An inherent risk is that regenerative agriculture will follow the same pattern, and come to be defined by specific standards or practices, rather than by the principles and goals of a regenerative system. There are a growing number of certification programs and definitions for regenerative farming that capture key themes of regenerative agriculture and assemble various practices that may produce intended outcomes ( AGW, 2020; Carbon_Underground, 2020; HMI, 2020; ROC, 2020; TerraGenesis, 2020). In the end, any labelling or certification system must be validated empirically to determine whether it results in the intended outcomes.

Regenerative agriculture needs to be defined by principles and supported by empirical data from farms displaying a wide range of management practices to ensure the implemented principles are achieving regenerative farming goals. The approach to defining a regenerative system must be transparent, simple, scalable, transferable, incorruptible, and replicable. Our team has created a series of questions for land managers that encapsulate the principles that drive regenerative agriculture as defined by the farmers and ranchers ( Fenster *et al.*, 2021; LaCanne & Lundgren, 2018; Pecenka & Lundgren, 2019; Schmid & Lundgren, 2021). Each answer receives a score, and higher scores are indicative of more regenerative operations. A regenerative matrix score is attained by summing the scored results to these questions. The first goal of this paper is to determine whether key response variables pertaining to soil health, biodiversity, ecosystem function, and farm performance ( Elevitch *et al.*, 2018; Schreefel *et al.*, 2020) scale with the assignment of scores from our regenerative matrix. Essentially, does a higher regenerative score in cropland and rangeland consistently scale positively with the desired outcomes of regenerative farming? The second goal of this paper is to determine whether there is a threshold score that can be used to distinguish between regenerative and conventional systems? If this matrix performs these functions, then this approach could be used to advance the field of regenerative agriculture by providing a clear, scalable and transferable approach to characterizing a regenerative farm. 

## Methods

Two independent scoring systems based on a narrow suite of practices were used to define regenerative cropland and rangeland systems. Three food systems were examined in this project, corn in the Upper Midwest ( LaCanne & Lundgren, 2018), almond orchards in California ( Fenster *et al.*, 2021), and rangelands of the Northern Plains ( Pecenka & Lundgren, 2019; Schmid & Lundgren, 2021). Farms included in these studies represented successful, established systems and were in their respective farming philosophies for at least three years. Some of the farms were targeted for their reputation as being leaders in regenerative farming, but the resulting fields and ranches in reality represented a wide continuum from very conventional to very regenerative farming systems.

We examined different suites of response variables that are considered representative of a regenerative system. In croplands ( Table 1), the elimination of tillage, maintaining ground cover through planting cover crops or fostering resident vegetation, planting hedgerows, and use of organic amendments (compost, manure, mulch, compost teas), and grazing, were all considered regenerative ( LaCanne & Lundgren, 2018; Soto *et al.*, 2021). Tillage, maintaining bare soil, and spraying synthetic insecticides, herbicides, fungicides, use of chemical fertilizers were all considered conventional practices. Regenerative practices were scored as 1, and conventional practices were scored as 0. In rangelands ( Table 2), management systems were defined by their stocking density, rotation frequency, the duration that the pasture rested following grazing, and use of ivermectin products. Cattle operations were categorized based upon their combination of these practices to form the different systems ( Colley *et al.*, 2019; Teague & Barnes, 2017). Use of ivermectin in the ranches was categorized as high (multiple applications during a year; scored as 0), low (single annual use, not applied during grazing period; scored as 1) and no ivermectin (scored as 2). Operations’ stocking densities (animal units [AU] per ha), were categorized as fewer than 5 animal units (AU) per ha (scored as 0), 5–10 AU per ha (scored as 1), and more than 10 AU per ha (scored as 2). Operations were categorized as having a rotation frequency of 30 d or more (scored 0), between 10–30 d (scored as 1), and less than 10 d (scored as 2). Finally, rest periods on ranches were scored as continuously grazed (no rest; scored as 0), allowed to rest 1 > and < 30 d (1), or >30 d (2) over a growing season. A simple questionnaire to obtain the necessary information to populate our matrices can be found as in *Extended data* ( Lundgren, 2021).

**Table 1.** A matrix of farm practices in cropland/orchards that can be used to distinguish regenerative and conventional farms. Shaded rows refer to corn farms in the Upper Midwest, and unshaded rows refer to almond orchards in California. Fungicide use, organic amendments, and field margins/hedgerows were not described in the cornfields. A ‘1’ was assigned for each practice if it was considered regenerative and ‘0’ if it was considered conventional. Eliminating synthetic fertilizers, herbicides, fungicides, insecticides, and tillage were considered regenerative. Including cover crops, organic amendments (e.g., composts, compost teas, etc), livestock integration, and diversified field margins and hedgerows were also considered regenerative practices.

Field locations (County or nearest town, State)	Fertilizers	Herbicides	Fungicides	Insecticides	Tillage	Cover crops	Field margins/ hedgerows	Organic amendments	Grazers	Composite score
Merced, CA	1	1	1	1	0	1	1	1	0	**7**
Butte, CA	1	1	0	1	1	1	1	1	0	**7**
Merced, CA	1	1	1	1	1	1	1	1	0	**8**
Merced, CA	1	1	1	1	1	1	1	1	0	**8**
Yolo, CA	1	1	0	1	0	1	1	1	1	**7**
Butte, CA	0	0	0	0	1	1	0	1	0	**3**
Butte, CA	0	0	0	0	1	0	0	0	0	**1**
Yolo, CA	0	0	0	0	1	0	0	0	0	**1**
Yolo, CA	0	0	0	0	1	0	0	0	0	**1**
Butte, CA	1	1	0	1	1	1	1	1	0	**7**
Merced, CA	1	1	0	1	1	1	0	1	0	**6**
Merced, CA	0	0	0	0	1	0	0	0	0	**1**
Yolo, CA	1	1	1	1	1	1	0	0	1	**7**
Merced, CA	0	0	0	0	1	0	0	1	0	**2**
Merced, CA	0	0	0	0	1	0	0	1	0	**2**
Merced, CA	0	0	0	0	1	1	0	1	0	**3**
Bladen, NE	0	0		0	0	0			0	**0**
York, NE	0	0		0	0	0			0	**0**
Bismarck, ND	0	0		0	1	0			0	**1**
Bismarck, ND	0	0		0	1	0			0	**1**
White, SD	0	0		0	0	0			0	**0**
Pipestone, MN	0	0		0	0	0			0	**0**
Toronto, SD	0	0		0	0	0			0	**0**
Gary, SD	0	0		0	0	0			0	**0**
Arlington, SD	0	0		0	0	0			0	**0**
Lake Norden, SD	0	0		0	0	0			0	**0**
Bladen, NE	1	0		1	1	1			0	**4**
York, NE	1	0		1	1	1			0	**4**
Bismarck, ND	0	1		1	1	1			1	**5**
Bismarck, ND	1	0		1	1	1			1	**5**
White, SD	1	1		1	0	1			0	**4**
Pipestone, MN	1	1		1	0	1			0	**4**
Toronto, SD	1	0		0	1	1			0	**3**
Gary, SD	1	1		1	0	1			1	**4**
Arlington, SD	1	0		1	1	1			1	**5**
Lake Norden, SD	1	0		1	1	1			0	**4**

**Table 2.** A matrix of farm practices in rangelands that can be used to distinguish regenerative and conventional ranches. Unshaded rows refer to ranches that were included in the dung invertebrate assessment. Shaded rows refer to ranches that were examined in the fecal parasite and plant community study.

Ranch location (Nearest town, State)	Ivermectin	Stocking density	Rotation frequency	Rest period	Composite matrix score
Bruce, SD	1	0	0	0	**1**
Castlewood, SD	1	2	1	2	**6**
Clear Lake, SD	2	2	2	2	**8**
Estelline, SD	1	1	1	2	**5**
Estelline, SD	0	1	0	0	**1**
Flandreau, SD	0	0	0	0	**0**
Gary, SD	2	2	2	2	**8**
Goodwin, SD	2	2	2	2	**8**
Madison, SD	0	1	1	2	**4**
Milbank, SD	1	1	1	2	**5**
Milbank, SD	0	0	0	2	**2**
Sioux Falls, SD	2	2	2	2	**8**
Summit, SD	1	1	1	2	**5**
Thomas, SD	1	1	2	2	**6**
Twin Brooks, SD	2	2	1	2	**7**
Volga, SD	0	0	0	0	**0**
Hayti, SD	0	0	0	0	**0**
Hayti, SD	0	0	0	0	**0**
Strandburg, SD	0	0	0	0	**0**
Estelline, SD	0	0	0	0	**0**
Watertown, SD	1	2	2	2	**7**
Hayti, SD	1	1	2	2	**6**
Estelline, SD	2	1	2	2	**7**
Gary, SD	2	1	2	2	**7**
Goodwin, SD	1	1	2	2	**6**
Tuttle, ND	1	0	0	0	**1**
Tuttle, ND	0	0	0	0	**0**
Wing, ND	0	0	0	0	**0**
Moffit, ND	1	0	0	0	**1**
Fort Rice, ND	2	2	2	2	**8**
ND7; Wing, ND	2	1	2	2	**7**
Bismarck, ND	2	2	2	2	**8**
Wing, ND	2	2	2	2	**8**
Moffit, ND	2	2	2	2	**8**
Summit, SD	0	0	2	2	**4**
Summit, SD	0	0	0	0	**0**
Milbank, SD	2	1	1	2	**6**
Milbank, SD	0	0	0	0	**0**
Watertown, SD	2	2	2	2	**8**
Castlewood, SD	0	0	0	0	**0**
Summit, SD	2	0	2	2	**6**
Milbank, SD	0	0	0	0	**0**
Castlewood, SD	2	2	2	2	**8**
Castlewood, SD	0	0	1	2	**2**
Sheldon, ND	0	1	2	2	**5**
Sheldon, ND	0	0	0	0	**0**
Napoleon, ND	0	2	2	2	**6**
Napoleon, ND	0	0	0	0	**0**
Ellendale, ND	2	2	2	2	**8**
Forbes, ND	1	0	1	2	**4**
Forbes, ND	2	2	2	2	**8**
Berlin, ND	0	1	1	0	**2**

Management practices of cattle operations were scored 0-2 based, with higher numbers reflecting practices that promote biodiversity and soil quality. Ivermectin application frequency was divided into multiple applications (0), single application not during grazing season (1), and no ivermectin use (2). Stocking density (animal units; AU) was divided into <5 AU/ha (0), 5-10 AU/ha (1), and >10 AU/ha (2). Rotation frequency was divided into >30 d rotation (0), 10-30 d rotation (1), and <10 d rotation (2). Rest period was considered as continuously grazed during the growing season (0), a rest period of 1-30 d (1), and a rest of > 30 d (2).

### The study systems

Corn was examined on 20 farms (each with four fields) in North Dakota, South Dakota, Minnesota, and Nebraska in 2015 and 2016. Fields were a minimum of 4 ha in size, and the sampling area in each field was 61 × 61 m. All samples were taken at least 12 m into the field to minimize border effects. Only three of these farms were certified organic. Genetically modified (Bt hybrid) corn was universally treated with neonicotinoid seed treatments and was regarded as insecticide-treated. In corn, we examined soil organic matter (SOM), fine particulate organic matter (fPOM), soil bulk density, water infiltration, invertebrate communities in the soil, on the soil surface and in the plant canopy, pest abundance, yields, and profits.

Sixteen California almond orchards were studied in 2018 and 2019. Replicate plots (n = 4; 40 × 40 m) were established in each orchard. Plots were established 20 m into the field to avoid field margin effects. The orchards in the study ranged from the Northern half of the San Joaquin Valley through the Capay Valley to Chico. Orchards were 3–38 y old. Almond orchards are generally planted with at least two different varieties, with over-lapping bloom periods to promote pollination. Therefore, all of the orchards in the study contained at least two varieties, and almond varieties varied among the orchards. In each orchard, we examined a full range of soil and water characteristics, soil microbial, plant, and invertebrate community characteristics, pest damage, yields, and economics.

Dung invertebrate communities were examined on 16 ranches in a 7,935 km ^2^ region in eastern South Dakota during 2015 and 2016. All sites were grazed by cattle for at least 5 y, but annual grazing intensity and grazing period varied. Herds ranged from 20 to 120 individuals, and the cattle differed in size, breed, and administered ivermectin products. Focal pastures were at least 4 ha in size. The systems were ranked from regenerative to conventional based on several practices ( Table 2). In our study, the designation of management systems was based upon the grouping of several management practices that were occasionally all present on the same cattle operation.

A second study of cattle ranches examined plant communities and helminth fecal parasites. Selected sites (n = 20 per grazing treatment) focused on rangelands in the Dakotas during 2019 and 2020. Grazing treatments were assigned based on a ranch management character matrix, which consisted of rancher-defined best management practices for regenerative and conventional ranching systems ( Table 2). This character matrix was adapted from a similar study conducted in the region by Pecenka & Lundgren (2019). Grazing treatments were paired within each region and year. Each grazing system had been practiced on the site for at least 4 years.

### Soil quality

Soil physical and chemical properties were assessed in corn fields and in almond orchards. SOM and bulk density (BD) were assessed in both of these systems. In cornfields and almonds, four soil cores (8.5 cm deep, 5 cm in diam) were randomly collected from a single field on each of 20 farms; cores were collected at locations that were at least 10 m apart. On each sample, vegetative material was removed from 60 g of soil by hand, and the resulting samples were stored in aluminum containers at 105°C overnight. SOM in the soil sample was measured using the weight loss on ignition (LOI) technique ( Davies, 1974). To measure bulk density, four soil cores of known volume per field were weighed before and after drying at 100°C for 55 h.

In cornfields, we also measured fine particulate organic matter (fPOM). Approximately 30 g of soil was weighed in sterile aluminum pans. The soil was soaked in 90 mL of hexametaphosphate for 24 h, mixed for 5 min with a stainless-steel mixer, and were then sieved through screens with 500 μm and then 53 μm holes; the finer screen isolated the fPOM fraction. The isolated sample was then weighed, dried for 24 h at 105°C. Samples were then cooled in a desiccator cabinet, weighed, baked in a furnace for 4 h at 450°C. The samples were then cooled in a desiccator cabinet and weighed a final time.

In almond orchards, soil samples were collected at random locations in the inter-row area of each plot to determine total soil carbon (TSC) and total soil nitrogen (TSN). The probe (2.54 cm × 91.44 cm) was inserted 60 cm deep. Each core was placed into a plastic bag that was stored on ice until it could be transferred to a paper bag in the laboratory. Samples were weighed, dried, and weighed again. All visible pieces of rock and organic matter were removed from the samples. The samples were ground and were then passed through a sieve with 0.180 mm openings. Elemental analysis was conducted on three subsamples (12–15 mg) that were housed on tin capsules (5 × 9 mm) (ECS 8020, NC Technologies, Milan, Italy). To control for the relative compaction and other circumstances, the mass (Mg) of TSC per ha was assessed using the Equivalent Soil Mass (ESM) method, in which a cubic spline of Mg of TSC per depth layer was calculated ( Wendt & Hauser, 2013). This resulted in the assessment of carbon as Mg of TSC/ha at 6,000 Mg (59.2 cm deep). 

Soil macro- and micronutrients were also quantified in the almond orchards. The samples were ground to pass a 2 mm sieve and divided into three subsamples (two were 4 g each and one weighed 40 g). The 40 g soil sample is analyzed with a 24 h incubation test at 24 ^o^C. This sample is wetted through capillary action by adding 20 mL of deionized water to a 237 mL glass jar and then capped. After 24 h, the gas inside the jar was analyzed using an infrared gas analyzer (IRGA) (Li-Cor 840A, LI-COR Biosciences, Lincoln NE) for CO _2_-C. The two 4 g samples were extracted with 40 mL of deionized water and 40 mL of H ^3^A to extract the NO _3_-N, NH _4_-N, and PO _4_-P from the samples. The samples were shaken for 10 min, centrifuged for 5 min, and filtered through filter paper (Whatman 2V, Cytiva, Marlborough, MA). The water and H ^3^A extracts were analyzed on a flow injection analyzer (Lachat 8000, Hach Company, Loveland CO). The water extract was also analyzed on a Teledyne-Tekmar Torch C:N analyzer for water-extractable organic C and total N. The H ^3^A extract was also analyzed on a Thermo Scientific ICP-OES instrument for P, K, Mg, Ca, Na, Zn, Fe, Mn, Cu, S and Al *.* The Haney soil health score combines five independent measurements of a soil’s biological properties to provide a general estimate of the overall health of a soil system ( Haney *et al.*, 2018). It is calculated as 1-d-CO _2_-Carbon/10 plus water extractable organic carbon (WEOC)/50 plus water extractable organic nitrogen (WEON)/10.

### Water infiltration

We used the rainfall infiltration rate kits of the Natural Resource Conservation Service (NRCS) to sample water infiltration rates in northern cornfields and California almonds. A metal ring (15 cm diam, 13.5 cm tall) was hammered 6.5 cm into the soil. Water (444 mL) was poured into the ring, and the time it required to saturate into the soil was recorded. A second container of 444 mL of water was poured into the ring, and the time to saturation was recorded again. In cornfields, this process was recorded during anthesis.

### Microbial diversity

Microbial communities were only sampled in almond orchards using the Phospholipid fatty acid (PLFA) test. Soil cores (15 cm depth, 1.9 cm diam; N = 16), were taken from four replicates per site per farm during the fruiting period. The samples were taken at random locations within each replicate, at least 5 m apart, using a transect that diagonally bisected the plot. Soil cores for each orchard were combined in a sealed plastic bag and placed in a cooler with dry ice ( Drenovsky *et al.*, 2010). Soil samples were stored at -80˚C until they could be freeze-dried and ground to 2 mm particle sizes. Phospholipid fatty acid (PLFA) testing provided an index of a soil’s microbial biomass and composition ( Frostegård *et al.*, 2011). The microbial biomass and community composition were recorded as total microbial biomass, undifferentiated microbial biomass, total bacteria, Gram-positive bacteria, Actinomycetes, Gram-negative bacteria, *Rhizobia* bacteria, total fungi, arbuscular mycorrhizal fungi, saprophytic fungi, and Protozoa.

### Plant community structure

Plant community structure was assessed solely as ground cover in the almond study. The ground cover height and composition in each of the replicates/plots was recorded during each of the three sampling periods. The percent ground cover was categorized as 0–25%, 25–50%, 50–75%, and 75–100%. Percent ground cover in the overall orchard was assessed using visual assessments of the percent ground cover in each invertebrate quadrat. 

Plant community diversity and green canopy cover were examined during the 2019 and 2020 grazing seasons of the rangeland study, while vegetation biomass was assessed only during the 2020 season. All three plant community metrics were measured during three periods of the grazing season; early June, mid-July, and late-August/early-September. At each pasture site, two 50 m transect lines were established 15 m apart and perpendicular to the slope of the land. Plant community diversity was assessed using the belt transect method for monitoring native prairie vegetation in the Dakotas ( Grant *et al.*, 2004). Briefly, plant community structure was recorded for a randomly selected 5 × 0.5 m ^2^ belt along each transect line. Vegetation is classified in each 5 m segment according to a hierarchical breakdown of plant groups common to the region ( Grant *et al.*, 2004). Green canopy cover was documented using the smartphone app Canopeo, which quantifies fractal green vegetation canopy cover area per unit area ( Patrignani & Oschner, 2015). Quadrats (50 × 25 cm ^2^) were established at 0, 25, and 50 m along transect lines to quantify green canopy cover with Canopeo. Lastly, a disc pasture meter was used to estimate above-ground vegetation biomass along each transect line and within their immediate surrounding area (<15 m). Compression height of vegetation was recorded by dropping a 0.13 m ^2^ plate weighing 1.34 kg from a height of 183 cm.

### Insect diversity

Insect surveys in cornfields and almonds involved sampling the epigeal community from 0.25 m ^2 ^sheet-metal quadrats (15 cm tall) inserted into the soil ( Lundgren *et al.*, 2006). Quadrats were placed in randomly selected locations between corn rows (n = 5 per plot) during anthesis. Invertebrates were exhaustively collected from the surface of the soil and beneath plant debris using handheld mouth aspirators. In almonds, sampling of the invertebrate communities occurred during the bloom, fruit development, and harvest periods. The invertebrate communities that could be collected from the soil surface and top 2 cm of the soil with mouth-operated aspirators in 15 min were preserved in 70% ethanol.

In cornfields, we also sampled the foliar invertebrate community. The foliar invertebrate community was sampled using a destructive whole plant assessment. Corn plants (n = 25 per plot) were randomly selected, thoroughly examined, and invertebrates that were not sight-identified were collected using mouth aspirators. Plants were then severed at the ground level and were transported to the field margin where their leaves, stems, ears, whorls, and tassels were inspected and dissected on white sheets. 

Soil invertebrate and dung invertebrate communities were assessed using soil cores in corn fields and rangelands. In corn fields, five soil cores (10 cm diameter, 10 cm height) were collected per field in randomly selected locations within and between corn rows during anthesis. In rangelands, the same core approach was used to collect invertebrates from 2–5 d old dung pats and the soil directly beneath the pat (n = 10 per ranch). Samples were collected monthly from May to September. All cores were refrigerated for < 36 h, and then were placed into a Berlese collection system over 7 d, when they reached a constant weight.

All extracted invertebrates were identified microscopically and cataloged; each unique specimen was identified to the lowest taxonomic level possible representing a functional morphospecies. Each morphospecies was placed into a trophic guild (coprophage, predator, parasitoid, herbivore, or pest) based upon previous descriptions of the biology of arthropod community in these systems. Voucher specimens are housed in the Mark F. Longfellow Biological Collection at Blue Dasher Farm, Estelline, SD, USA.

### Pests

In cornfields, pests included aphids, lepidopteran larvae, and corn rootworm adults that were identified from the foliar bioinventories. In almonds, pest incidence was assessed on 500 almonds per farm in 2018 and 600 almonds per farm in 2019 (< 20 from any one tree) ( Bentley *et al.*, 2001; Doll, 2009; Legner & Gordh, 1992). The almonds were each categorized as having no pest damage, navel orange worm damage ( *Amyelois transitella* [Walker] *;* Lepidoptera: Pyralidae *),* ant damage (Formicidae *),* oriental fruit moth damage ( *Grapholita molesta* [Busck]; Lepidoptera: Tortricidae), peach twig borer damage ( *Anarsia lineatella* Zeller; Lepidoptera: Gelechiidae), leaf footed plant bug or stinkbug damage (Hemiptera: Coreidae, Pentatomidae), and unknown pest damage ( Bentley *et al.*, 2001; Doll, 2009; Symmes, 2018).

Fecal oocyst counts of coccidia and egg counts of gastrointestinal nematodes belonging to the superfamilies Trichostrongyloidea and Strongyloidea were conducted on herds from the 2019 – 2020 rangeland study. Three grams of dung was collected from 2 – 4 d old cattle dung pats present in pastures (n = 5 pats sampled/site). Fecal samples were processed according to the Modified Wisconsin Sugar Float Technique ( Cox & Todd, 1962). Coccidia oocysts and eggs of Trichostrongyles and Strongyles were quantified for 1 g of fecal sample. Sampling for internal parasites in the herds was conducted twice in 2019 (mid-July and late-August/early September) and three times in 2020 (early-June, mid-July, and late-August/early September). 

### Yield and profit

In cornfields and almonds, responses from producer surveys were used to determine management practices, costs, and revenues that went into the direct net profitability of each operation. Gross profit analysis included yield, return on grain, and additional revenue that included livestock grazing and production. In cornfields, yield information provided by the farmers were confirmed by yields that were hand-gathered from three 3.5-m row sections from each replicate-field. Costs associated with corn production included corn seed, cover crop seed, drying/cleaning grain, crop insurance, tillage, planting corn, planting cover crop, fertilizers, herbicides, and irrigation (additional information on profit/loss analysis can be found in LaCanne & Lundgren 2018). Operating costs in almonds included winter sanitation, sampling for tree nutrient status and soil salinity, pH, and nutrient levels, irrigation and frost protection, fertilizers, insecticides, herbicides, fungicides, disease treatment sprays, trapping vertebrate pests, cover crop seed, tillage, mowing, flamers, grazers, and harvest.). Harvest in the almonds included the hourly labor to conduct the harvest and the price paid to external contractors (additional information on the almond profit/loss analysis can be found in Fenster *et al*. in prep). In both systems, only direct costs and revenues were used to calculate profitability.

### Data analysis

Unless otherwise described, we used linear regressions to search for correlations between the regenerative matrix scores and response variables. In the almond study, clay percentages of the soils were considered as co-factors in all models that examined TSC. Plot values were composited across plots into single values in the almond study and linear regression analyses were used to compare regenerative scores and response variables. In instances where resampling a single farm was performed (either spatially or temporally) Linear Mixed Models were used to remove pseudoreplication and account for this dependence; field and farm were included as random factors in the corn models, and month was included as a fixed factor and farm as a random factor in the rangeland studies. All statistics were conducted using Systat 13.1 (Systat Software Inc., San Jose, CA)

## Results

### Soil physical and chemical properties

**Figure 1.**
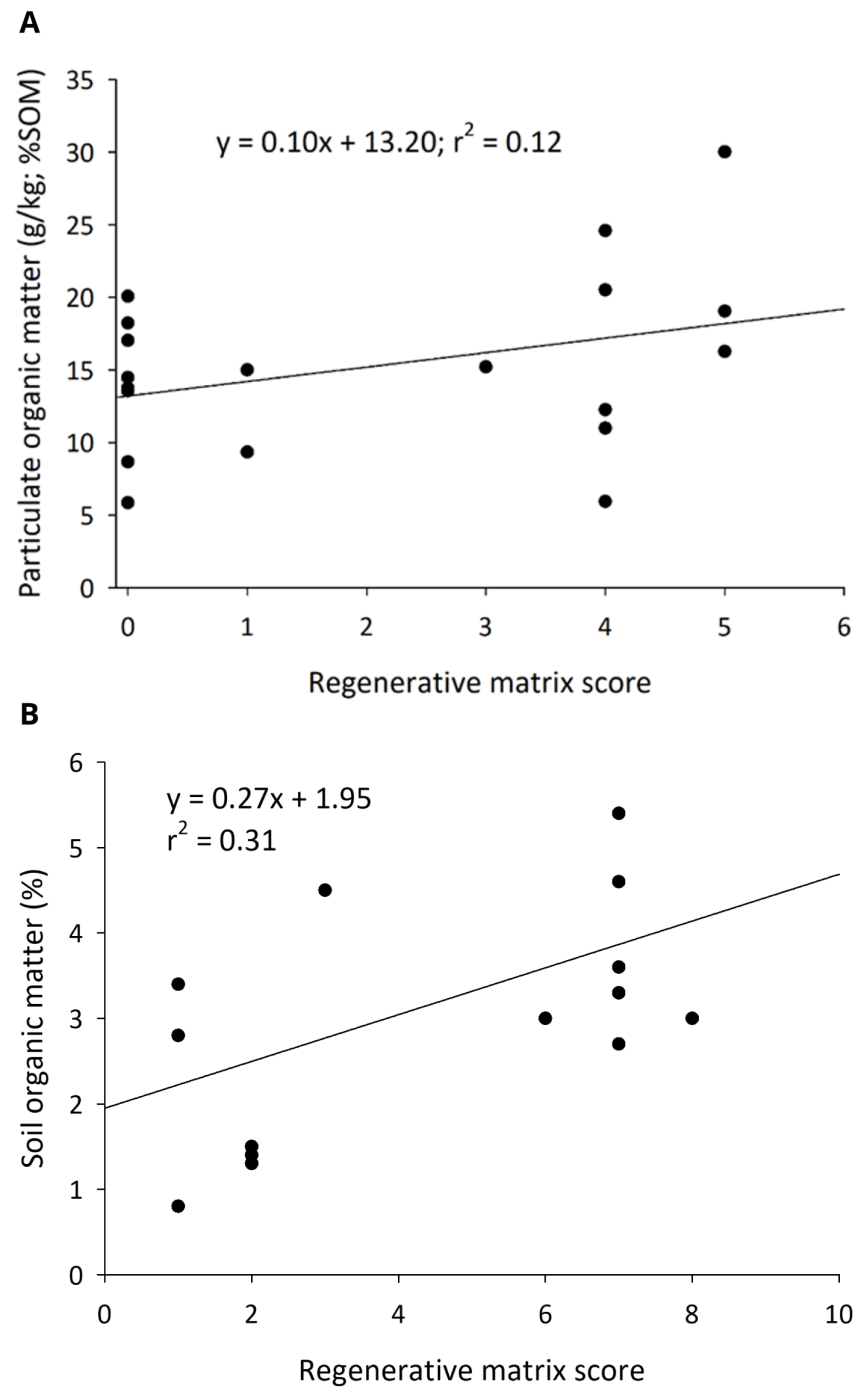
The relationships of regenerative farming intensity on fine particulate organic matter in cornfields of the Upper Midwest ( **A**) and percent soil organic matter in California almond orchards ( **B**).

In cornfields, fPOM of the soil was strongly and positively associated with regenerative matrix scores (F _1, 17 _= 4.61; *P* = 0.047) ( Figure 1A), but SOM (F _1, 17 _= 0.11; *P* = 0.75), bulk density (F _1, 17 _= 2.32; *P* = 0.15), and water infiltration rates (F _1, 11 _= 0.47; *P* = 0.51) of the soil were not correlated with matrix scores. The slowest water infiltration rates in the higher regenerative scoring farms were those that practiced tillage. 

**Figure 2.**
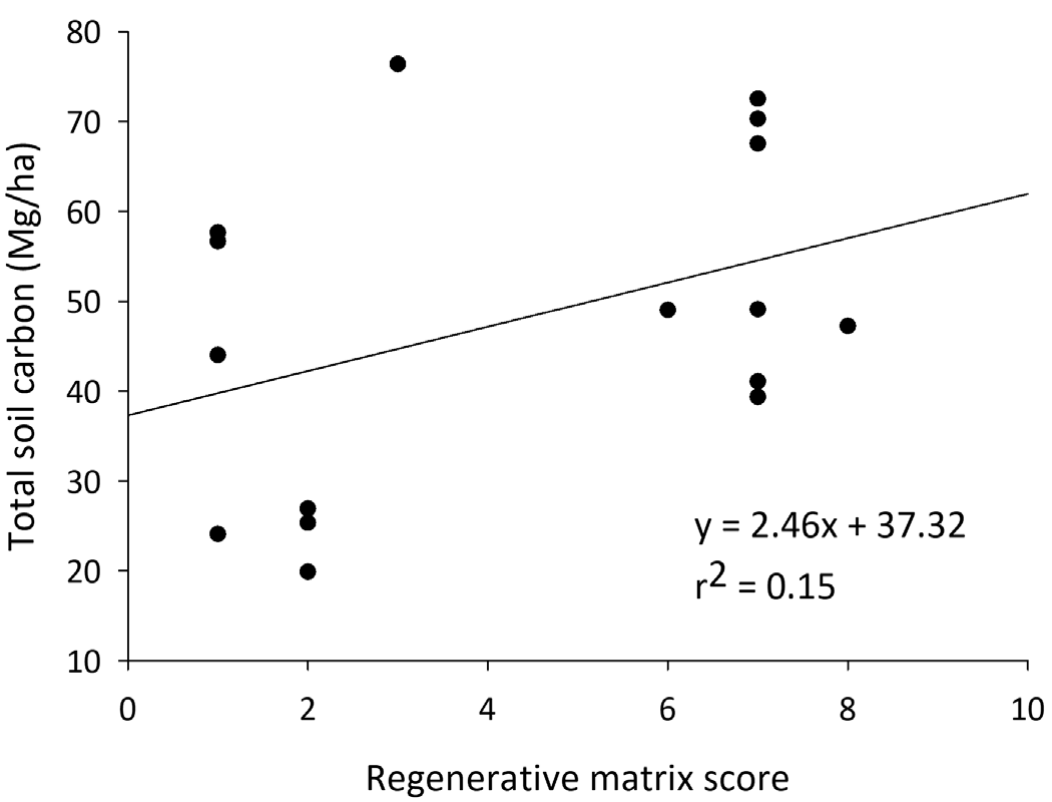
Total soil carbon in California almond orchards as the orchards increase the number of regenerative practices.

In almond orchards, higher matrix scores correlated to significantly higher levels of TSC and TSN (500–6000 ESM layers) and SOM (15 cm depth). At the 0–6000 Mg ESM layer, there was a significant correlation between the orchards’ matrix scores and TSC (model: F _2, 13 _= 13.77, *P* = 0.001; score: t = 2.26, *P* = 0.04; clay: t = 4.73, *P* < 0.001) ( Figure 2). At the 6000 Mg ESM layer there was a significant correlation between the orchards’ matrix scores and TSN (model: F _2, 13 _= 17.73, *P* < 0.001; score: t = 1.94, *P* = 0.08; clay: t = 5.63, *P* < 0.001). In contrast to what we found in cornfields, in the top 15 cm of soil there was a significant correlation between the orchards’ SOM%, matrix scores, and clay percentage (model: F _2, 13 _= 47.56, *P* < 0.001; score: t = 5.50, *P* < 0.001; clay: t = 8.05, *P* < 0.001) ( Figure 1B).

In almonds, higher matrix scores and soil clay percentages correlated to higher levels of WEON (F _2, 13_ = 9.16, *P* = 0.003) and WEOC (F _2, 13_ = 15.61, *P* = 0.001) in the organic matter of the soils. Total phosphorus (model: F _2, 13 _= 4.32, *P* = 0.04; score: t = 2.65, *P* = 0. 02, clay: t = -1.28, *P* = 0.23) and inorganic phosphorus (model: F _2, 13_ = 4.56, *P* = 0.03, score: t = 2.74, *P* = 0.02, clay: t = -1.28, *P* = 0.22) were significantly and positively correlated with an orchard’s matrix score. Higher matrix scores correlated to higher levels of Calcium (F _2, 13_ = 5.97, *P* = 0.01) and Sulfur (F _1, 14 _= 10.80, *P* = 0.01), but lower levels of Aluminum (F _1, 14 _= 6.13, *P* = 0.03). There were no correlations between other micronutrients and the regenerative matrix score ( *P* > 0.05). Haney soil health test scores were positively correlated with regenerative matrix scores (model: F _2, 13 _= 7.82, *P* = 0.01, Score t = 2.99, *P* = 0.01, clay: t = 2.59, *P* = 0.02) ( Figure 4). Soil respiration was unaffected by regenerative matrix scores (model: F _2, 13 _= 2.91, *P* = 0.09, Score t = 1.62, *P* = 0.13, clay: t = 1.77, *P* = 0.10).

**Figure 3.**
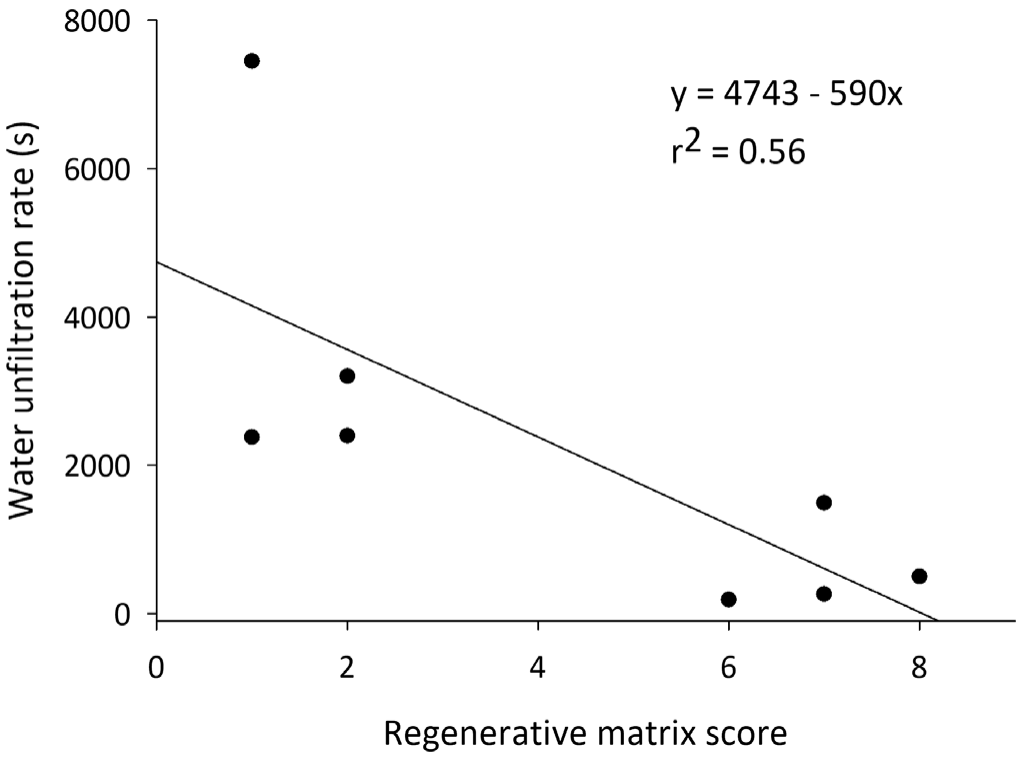
Water infiltration rates in the soils of California almond orchards in relation to the number of regenerative practices on each orchard.

**Figure 4.**
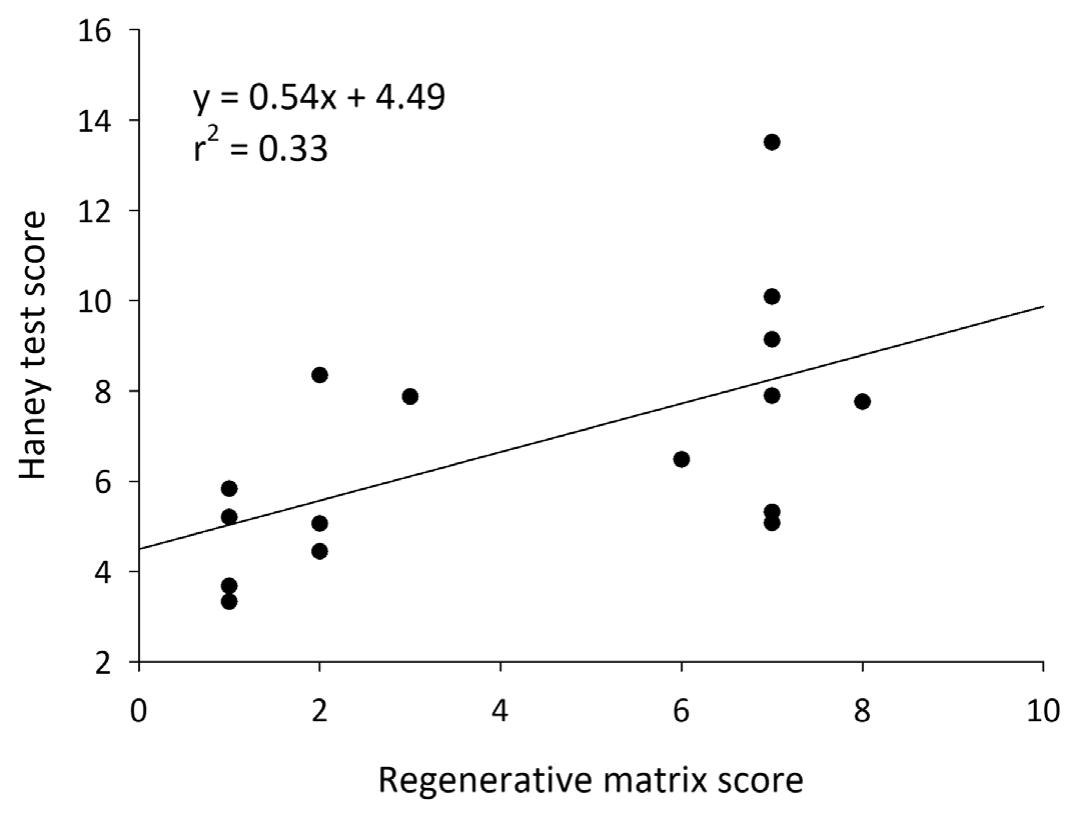
Haney test scores on soils from California almond orchards with increasing numbers of regenerative practices.

Higher regenerative-conventional matrix scores in almonds correlated to lower bulk densities (model F _2, 13 _= 8.95, *P* = 0.004; score: t = -3.91, *P* = 0.002, clay: t = -1.61, *P* = 0.13). Water infiltration rates increased alongside matrix scores for these orchards (model: F _2, 5_ = 11.43, *P* = 0.01; score: t = -4.50; *P* = 0.009, clay: t = 2.56, *P* = 0.05) ( Figure 3).

### Soil microbiology

In almond orchards, higher matrix scores correlated to higher amounts of bacterial biomass (F _1, 14 _= 6.25, *P* = 0.03) and higher Gram (+) biomass (F _1, 14 _= 9.12, *P* = 0.01). The percent composition in the microbial community was not affected by regenerative score (F _1, 14 _= 2.47, *P* = 0.14), indicating greater overall microbial biomass rather than bacterial dominance. The remainder of these microbial community metrics were not correlated to the regenerative conventional matrix score ( *P* > 0.05).

### Plant community

**Figure 5.**
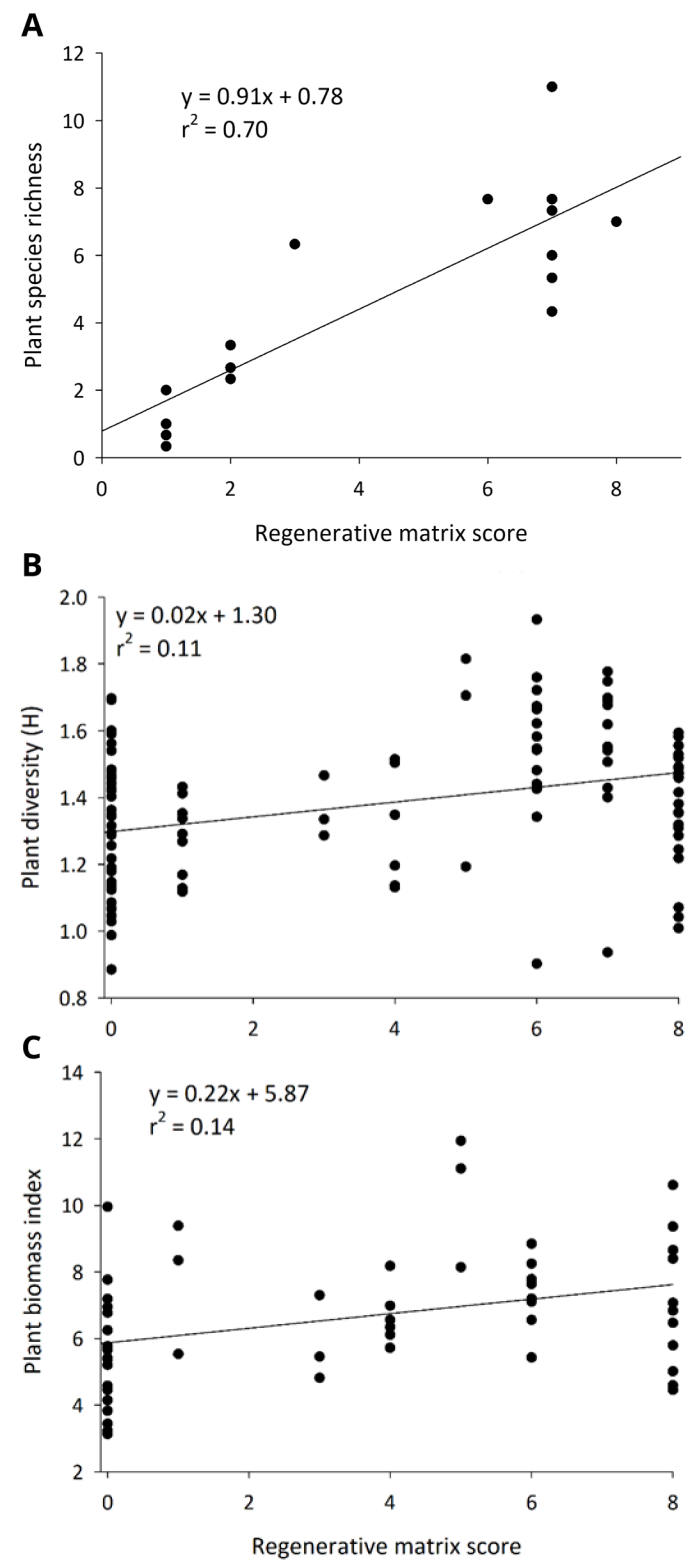
Plant species richness on the orchard floor in California almonds ( **A**), and plant diversity (Shannon H; **B**) and biomass index ( **C**) in rangelands of the Northern Plains as they relate to how regenerative a farm is.

More regenerative cropland and rangeland supported more diverse and robust plant communities. In almond orchards, higher matrix scores correlated to more ground cover (F _1, 14 _= 127, *P* < 0.001), more plant species (F _1, 14 _= 27.61, *P* < 0.001) ( Figure 5A), and greater biomass height (F _1, 14 _= 9.90, *P* = 0. 01). In rangelands of the Northern Plains, there was a significant relationship between matrix score and plant biomass (Score: F _1, 53 _= 8.47, *P* = 0.01; month: F _2, 53 _= 0.45, *P* = 0.64) ( Figure 5B), plant diversity (Score: F _1, 106 _= 14.22, *P* < 0.001; month: F _3, 106 _= 1.02, *P* = 0.39) ( Figure 5C), and ground cover (Score: F _1, 106 _= 10.06, *P* = 0.002; month: F _3, 106 _= 14.76, *P* < 0.001).

### Invertebrate community

**Figure 6.**
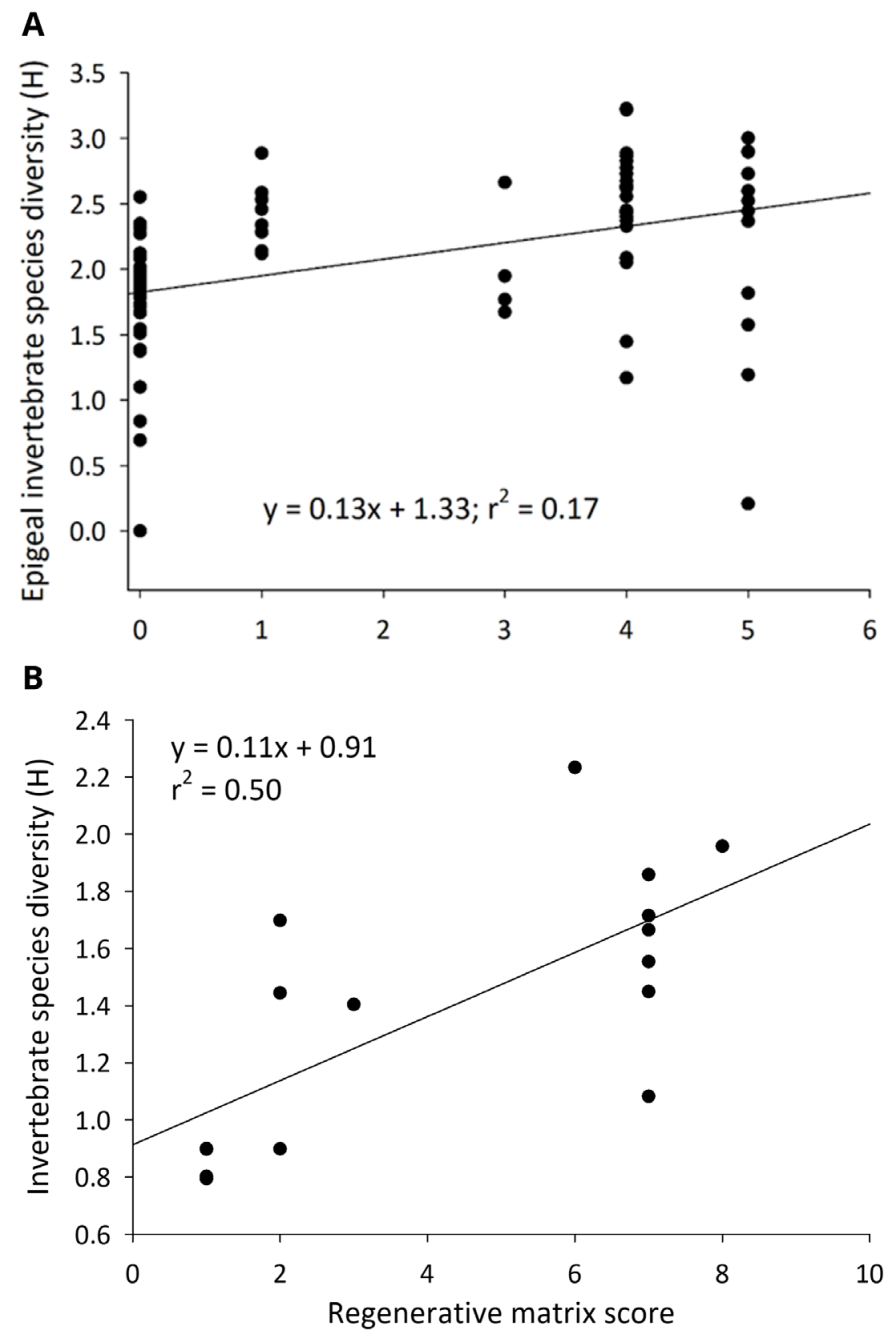
The invertebrate species diversity (Shannon H) on the soil surface in cornfields of the Upper Midwest ( **A**), and on the orchard floors of California almonds ( **B**), as they relate to the number of regenerative practices implemented on farms.

Across cropland and rangeland, more regenerative farms had more robust invertebrate communities. Several aspects of invertebrate community structure in cornfields were strongly and positively correlated with how regenerative a field was scored. The abundance (F _1, 55 _= 6.53, *P* = 0.01), species richness (F _1, 55 _= 38.31, *P* < 0.001), and diversity (F _1, 55 _= 7.34, *P* = 0.01) ( Figure 6A) of the epigeal invertebrate community was positively affected by matrix score. Similarly, matrix scores were positively associated with the species richness of the invertebrate community found in the soil column (F _1, 56 _= 12.96, *P* = 0.001), but the abundance was only marginally associated with matrix score (F _1, 56 _= 3.41, *P* = 0.07), and the diversity of this community was unaffected by matrix score (F _1, 56 _= 2.45, *P* = 0.12). Foliar invertebrate abundance (F _1, 55 _= 0.99, *P* = 0.32), species richness (F _1, 55 _= 1.35, *P* = 0.25), and species diversity (F _1, 55 _= 0.20, *P* = 0.66) were not related to the matrix scores.

The invertebrate community on the soil surface was positively affected by the number of regenerative practices on almond orchards. Invertebrate abundance (F _1, 14_ = 10.28, *P* = 0.01), biomass (F _1, 14 _= 70.10, *P* < 0.001), species richness (F _1, 14_ = 14.44, *P* = 0.002) and species diversity indices (H: F _1, 14_ = 12.37, *P* = 0.003; DS: F _1, 14_ = 11.11, *P* = 0.01) ( Figure 6B) were positively correlated with matrix score. There was a positive correlation between earthworm (square rooted to normalize residuals) abundance and matrix score on these orchards (F _1, 14_ = 9.87, *P* = 0.007).

**Figure 7.**
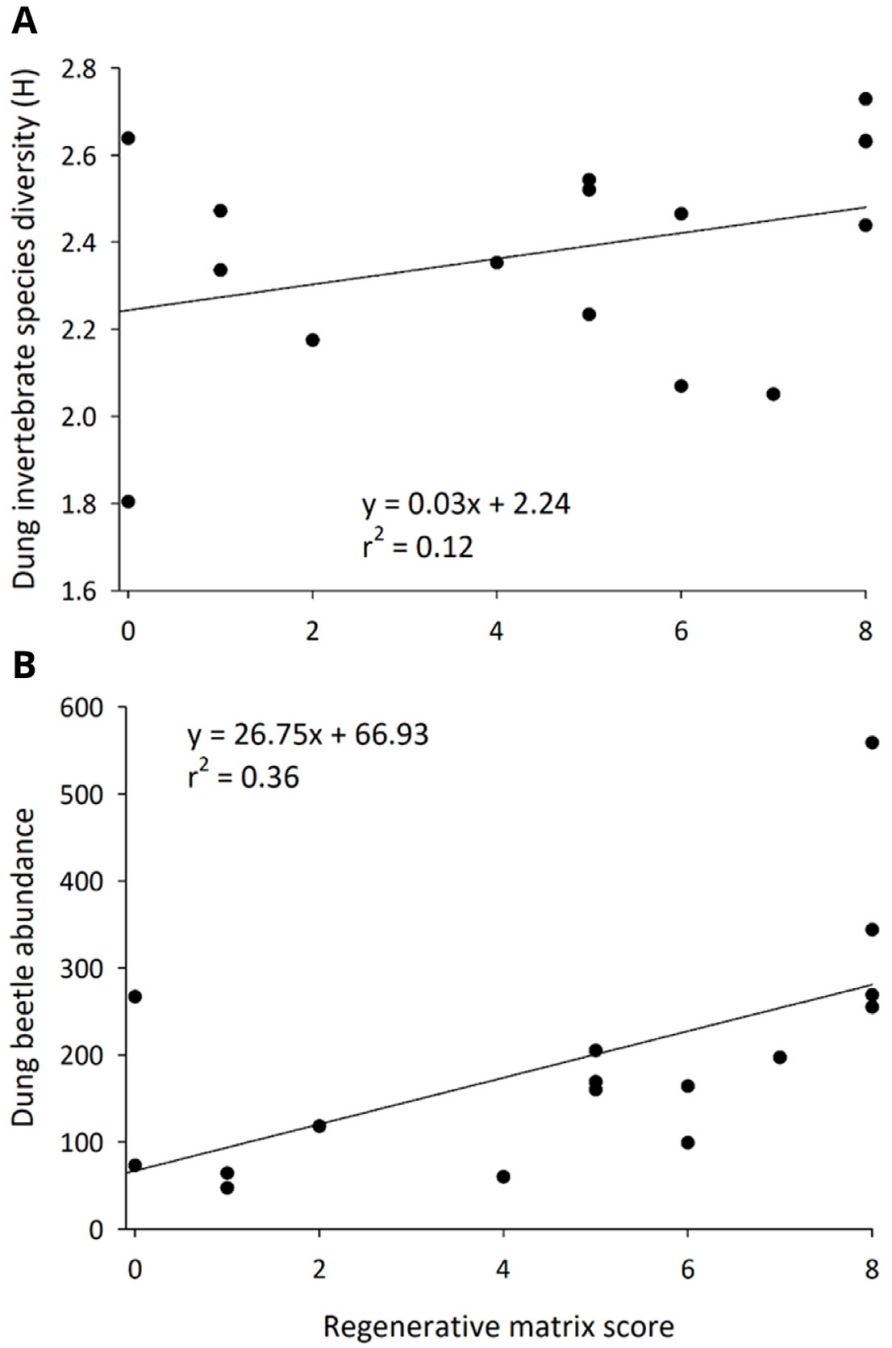
Invertebrate species diversity (Shannon H) in dung pats ( **A**) and the abundance of dung beetles ( **B**) as they relate to regenerative intensity on ranches in the Northern Plains.

In rangelands of the Northern Plains, the invertebrate community associated with cattle dung was strongly and positively associated with the matrix score attained. The species richness (Score F _1, 74 _= 15.38, *P* < 0.001; month F _4, 74 _= 6.54, *P* < 0.001), and species diversity (Score F _1, 74 _= 16.10, *P* < 0.001; Date: F _4, 74 _= 1.50, *P* = 0.21) ( Figure 7A) of the dung invertebrate community were positively associated with matrix score. Abundance of dung invertebrates was uncorrelated with matrix score (Score F _1, 74 _= 0.01, *P* = 0.99; month: F _4, 74 _= 4.83, *P* = 0.001). The abundances of two critical functional groups, dung beetles (Score F _1, 60 _= 10.05, *P* = 0. 006; month: F _4, 60 _= 5.70, *P* = 0.001) ( Figure 7B) and invertebrate predators, (Score F _1, 74 _= 7.41, *P* = 0.01; month: F _4, 74 _= 4.09; *P* = 0.01) increased as a rangeland became more regenerative.

### Pest populations

In all of these systems, pest populations were uniformly below any recognized economic thresholds. Pests were not correlated with the number of regenerative practices used in cornfields (pest abundance: F _1, 55 _= 2.18, *P* = 0.15) or in almond orchards (% damaged nuts: F _1, 14 _= 0.69; *P* = 0.42). In rangelands, there was no relationship between the matrix score and *Trichostrongyles* (Score: F _1, 88 _= 0.09, *P* =0.77; month: F _3, 88 _= 0.39, *P* = 0.76) or *Coccidia* (Score: F _1, 88 _= 1.78, *P* = 0.19; month: F _3, 88 _= 0.70, *P* = 0.56) fecal parasite loads.

### Yields

Corn yields were negatively correlated with the regenerative matrix score (F _1, 56 _= 3.88, *P* = 0.05). Despite this, three of the top ten yielding cornfields were regenerative, indicating that regenerative practices are not necessarily tied with yield reductions. Standard deviations became greater proportions of the mean as a farm became more regenerative (variability in yields were higher among regenerative farms) (R ^2^ = 0.79; F _1, 4 _= 5.12, *P* = 0.11). In almonds there was no relationship between almond yield and how regenerative an orchard was (F _1, 12_ = 0.86, *P* = 0.37). On a treatment level, regenerative farms had twice the variance in yield relative to the conventional orchards.

### Economics

**Figure 8.**
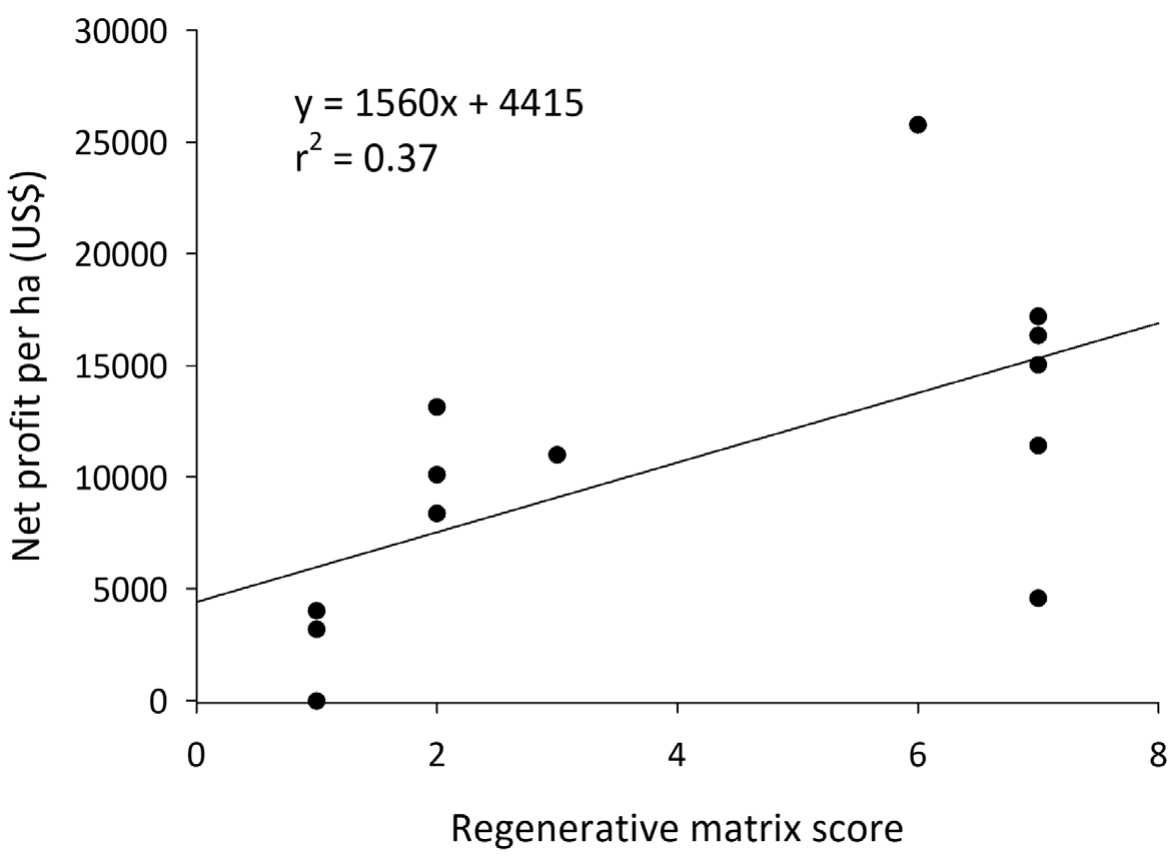
Net profit of California almonds as it relates to the number of regenerative practices on orchards.

Although there was a positive relationship between matrix score and profitability in cornfields, this relationship was not significant (F _1, 53 _= 2.49, *P* = 0.12). In cornfields, there was a significant increase in the standard deviation of profit as a farm became more regenerative (e.g., standard deviation as a proportion of the mean; R ^2^ = 0.72; F _1, 4 _= 7.68, *P* = 0.07). This indicates more variability in profits among the more regenerative producers. The top ten highest netting farms were all regenerative farms (scores of four and five). In almonds, profit was strongly and positively correlated with matrix scores (F _1, 11_ = 7.41, *P* = 0.02) ( Figure 8). Operating costs were unaffected by matrix score (F _1, 13 _= 0.70, *P* = 0.42), but net income increased as regenerative scores increased (F _1, 11_ = 8.50, *P* = 0.01).

## Discussion

Our approach to defining farms based on a small suite of carefully chosen practices was strongly associated with several key metrics that define regenerative systems, including soil health, biodiversity promotion and profitability. With our matrix, we have distilled complex agricultural systems down to fewer than 10 practices that serve as useful indicators of regenerative operations in cropland and rangeland. These practices represent each of the five principles that define regenerative agriculture in cropland and in rangeland. We have determined that these practices are regenerative across cropping systems; the cropland matrix works in row crop systems as well as a perennial system including orchards. Also, it was noteworthy that none of the regenerative farm attributes attained an asymptote, which suggests that farms could become more regenerative if they continue to incorporate additional regenerative practices. This matrix can be used to categorize a farm as regenerative or conventional based on a threshold score.

Regenerative farming practices inherently increase plant biomass and diversity in both cropland and rangeland systems, and this plant community is a central mechanism for improving the soil health, biodiversity, and resilience of these farming operations. Farmers in this study enhanced their plant diversity and biomass ( Figure 5) using a variety of tools. In almonds and cornfields, many of the farmers planted annual cover crops to improve the health of their soils. In some regenerative orchards, farmers allowed the native vegetation to persist. All of the regenerative orchards stopped the use of synthetic herbicides, and this likely contributed to greater plant diversity. In rangelands, short-term, intense grazing stimulates plant communities to diversify and grow if the plant community is allowed to rest following a grazing event ( Hillenbrand *et al.*, 2019; Teague *et al.*, 2011). Plants are a vital part of the carbon cycle, and as such they are the primary means whereby energy enters into an ecosystem ( Weil & Brady, 2017). Additionally, plant communities are requisite directly and indirectly to the genesis of healthy soils ( Lucas, 2001). Plant roots provide the needed polysaccharides for microbial communities to grow and perform key nutrient cycling services ( Philippot *et al.*, 2013). Physical properties of soils are also influenced by plants, including water infiltration rates, soil aggregate structure, bulk density, etc. ( Gulick *et al.*, 1994; Liu *et al.*, 2005). Plant diversity and biomass scales positively with the diversity of nearly every other group of organisms ( Bianchi *et al.*, 2006; Saunders *et al.*, 2013; Zak *et al.*, 2003), and thus plant communities are a driver of biodiversity within agricultural habitats. Taken in sum, the relationships between plant communities and regenerative score illustrate an important mechanism for how regenerative farms positively enhance many intended regenerative outcomes. 

Animal integration is another important tool for managing farms that improve regenerative outcomes. The most regenerative cropping operations observed in this set of studies always integrated livestock (chickens, sheep, or cattle), and these farms also had the greatest biodiversity, soil health, water infiltration rates, and economic metrics. Regeneratively-managed livestock in cropland improve soil chemical and physical properties, increase niche diversity which allows other organisms to thrive, and provides additional revenue and an additional plant management tool that can increase farm resilience ( Colley *et al.*, 2019; Delgado *et al.*, 2011; Niles *et al.*, 2018; Teague *et al.*, 2016). Important logistical constraints influence the circumstances of integrating livestock into cropland, especially in standing crops like almonds. Barriers preventing farmers from integrating livestock into cropland should be removed so this important management tool is more commonly adopted.

Soil chemical and physical properties were positively affected by the number of regenerative practices in cropland. Bulk density was significantly lower in the more regenerative almond orchards. Soil carbon and soil organic matter were strongly associated with more regenerative farms. Although we did not conduct a carbon life cycle assessment on corn or almonds, these results support the idea that regenerative farming systems can help our cropland sequester more carbon and help to offset greenhouse gas emissions ( Lal, 2020; Soto *et al.*, 2021). Organic and inorganic phosphorous are also enhanced as almond orchards become more regenerative. Phosphorous, a mined agricultural nutrient, is becoming increasingly rare and is available at significant economic and environmental costs ( Alewell *et al.*, 2020), so enhancing the plant-available forms of phosphorous that are present in the soils is an important outcome of regenerative agriculture. Micronutrients calcium and sulfur were also enhanced by increasing the number of regenerative practices in almonds. In almonds, water infiltration rates were significantly improved by regenerative production practices. Water is becoming more scarce ( Burri *et al.*, 2019), especially in California ( Mall & Herman, 2019), as climates continue to change and ground and surface waters are exhausted from conventional agricultural practices. Absorbing water into the soil when it becomes available is a crucial step in keeping these agricultural areas productive. Similar to previous studies ( Pikul & Aase, 2003; Pikul *et al.*, 2009), tilled cornfields had lower water infiltration rates and lower bulk density. This data supports the argument that no-till practices are an indispensable core component of regenerative agriculture, and argues in favor of mandating that regenerative operations be no-till.

All organismal groups measured were enhanced as farms became more regenerative. There was greater bacterial biomass and Gram+ bacteria from the farms with higher regenerative scores. In general, bacterial dominated soils are undesirable, as they tend to drive faster decomposition and reduce soil organic matter ( Hendrix *et al.*, 1986; Lehman *et al.*, 2015). Bacteria dominated soils are produced through tillage, and by definition, regenerative farms are not tilled. Only two of the almond orchards in this study had been tilled; and this must be considered when comparing bacterial communities in regenerative versus conventional almond systems. Despite increasing bacterial biomass in the regenerative orchard soils, the proportion of bacteria to other microbial life did not increase. Furthermore our soil health metric, the Haney Test that accounts for microbial function in a soil ( Haney *et al.*, 2018), was positively associated with the regenerative score of a farm. This suggests that although soil bacterial biomass may be increasing on regenerative almond orchards, it is not at the expense of other microbial taxa and it enhances bacterial function. Invertebrate biomass, diversity, and abundance in the soil, on the soil surface, and in the vegetation were positively affected by regenerative practices in all three study systems. This was particularly true for the epigeal communities. We hypothesize that promotion of the invertebrate community on the soil surface is a product of enhancing the plant community in this same stratum. Herbicides ( Bohnenblust *et al.*, 2016; Morton *et al.*, 1972), fungicides ( Johnson *et al.*, 2013), insecticides ( Bredeson & Lundgren, 2018; Pecenka & Lundgren, 2019; Seagraves & Lundgren, 2012), and synthetic fertilizers ( Cuesta *et al.*, 2008; Kromp, 1999) disrupt invertebrate communities. Regenerative farms that abandon these chemicals may experience enhanced invertebrate communities and biological diversity, which is important for farmers because it provides valuable services, including pest suppression. 

Pest populations of rangelands and croplands were below economically damaging levels, but these populations were suppressed using very different means in regenerative and conventional systems. In conventional systems, pests are kept low through genetically modified crops and insecticide applications. Conventional almond orchards in this study experienced up to five insecticide applications annually. All of the conventional corn farms are planted with genetically modified, insect resistant (i.e., Bt) varieties that were treated with neonicotinoids. All of the most conventional ranches applied ivermectins in their animals to reduce fecal parasite loads. Our research shows that regenerative practices produce the same low pest populations as when cattle were treated with ivermectins, but with lower input costs. Other work in agricultural ecosystems has shown that invertebrate diversity is essential to mitigating pest populations ( Crowder *et al.*, 2010; Letourneau *et al.*, 2009; Lundgren & Fausti, 2015; Lundgren & Fergen, 2014). This diversity within agricultural systems results in biological control performed by invertebrates and pathogens keeping pest populations at sub-economically damaging levels. We will test whether this same pattern was true in dung pats. Predation isn’t the only mechanism at work, and we also hypothesize that host plants and livestock are healthier in regenerative systems, which contributes to fewer pests ( Barbosa *et al.*, 2009). Also, in almond orchards the more robust epigeal invertebrate community or livestock may be playing a key role in breaking down fallen mummy nuts in which navel orange worm larvae overwinter. There also are likely synergisms among organisms in regenerative systems that balance communities and resist outbreaks of specific pest populations. But in the end, the complexity of biological network interactions in complex ecosystems prevent us from understanding all of the mechanisms whereby regenerative systems consistently suppress pests without pesticide inputs.

Improved soil health and biodiversity on the studied farms was associated with increased profitability. In almonds, there was a clear relationship between more regenerative practices and the profitability of the orchards. In cornfields, the most regenerative farms were also the most profitable, but there was substantially more variance in this relationship. These higher net profits were the result of lowering seed costs, reducing chemical premiums, and value-added marketing of the most regenerative products. Yields did not follow the same pattern as the profitability with regards to matrix scores. Almond yield was not correlated with how regenerative a farm was, and corn yields were significantly reduced as regenerative practices were added to cornfields. It is important to interpret these results recognizing that regenerative agriculture is still in its infancy in many production systems. Three of the top 10 yielding corn fields were regenerative, meaning regenerative corn production methods do not necessarily lead to yield reductions. Given the low reliance on corn as a human food source, and the lack of yield reductions in almonds, concerns over regenerative food systems’ ability to feed a growing human population are not supported by our research. All of this is to say that regenerative farming practices are correlated with increased profitability of a farm, thus supporting the notion that we can improve farm resilience while promoting the natural resource base of a habitat ( Schipanski *et al.*, 2016).

In looking at the data on these established farm systems, there were consistently two distinct clusters of farms based on regenerative score that could be used to categorize regenerative and conventional farming operations. The natural groupings could be divided at scores of 5 and 3 for cropland and rangeland, respectively (in cornfields, this dividing line was around 3, since we did not ask all of the questions to corn farmers in that study). One explanation for this may be that farms have trouble staying in business when they only employ an intermediate number of regenerative practices. Farms in the “regenerative” and “conventional” categories are not all created equal, since changing the number of regenerative or conventional practices can produce a variance in farm performance within each category. Also, the range of scores in the regenerative ranches were greater than that for in cropland, and could indicate a transition in the adoption of regenerative practices by ranchers of the Northern Plains. The duration that a farm is in its respective system also will likely affect the observed regenerative outcomes, and this temporal factor should be considered in further interpretations of regenerative scores. Uses for this matrix might include regulation based on carbon sequestration, water use efficiency, or pollution. Certification of regenerative operations and marketing of regenerative labelling might also employ the matrix approach as we have laid out. In the *Extended data*, we provide a survey for producers to generate their regenerative scores. We hope that this work will be built upon and tested as we confront the challenge of how to empirically define regenerative farming systems.

## Data availability

### Underlying data

Open Science Framework: Matrix paper, https://doi.org/10.17605/OSF.IO/G697Y ( Lundgren, 2021) (registered 15 ^th^ January 2021 https://osf.io/7v4z2).

This project contains the following underlying data:

- Corn
- Regenerative Rangeland 1
- Almond
- Regenerative Rangeland 2

### Extended data

Open Science Framework: Matrix paper, https://doi.org/10.17605/OSF.IO/G697Y ( Lundgren, 2021) (registered 15 ^th^ January 2021 https://osf.io/7v4z2).

This project contains the following extended data:

- CROPLAND Regenerative Score Calculator
- RANGELAND Regenerative Score Calculator

Data are available under the terms of the Creative Commons Zero "No rights reserved" data waiver (CC0 1.0 Public domain dedication).
