# Supplementary material for: Defining and validating regenerative farm systems using a composite of ranked agricultural practices
Source: F1000Res. 2021 Feb 15;10:115. [Version 1] doi: 10.12688/f1000research.28450.1 (PMC7953916; doi:10.12688/f1000research.28450.1)
Supplement: Peer review history

# Peer review: Reviewer response for version 1

**DOI:** 10.5256/f1000research.31489.r79612

**Published:** 2021-03-10

**Authors:** Richard W. Teague

**Affiliations:** 1 Texas A&M AgriLife Research, Vernon, TX, USA

This review of regenerative agriculture in many parts of US is the most comprehensive and thorough I have yet seen on the subject. It covers a wide array of biological disciplines to assess the various biodiversity elements required to assess the efficacy of regenerative agriculture relative to conventional agriculture.

 The authors are to be particularly commended for the solid statistical analyses that provide an excellent foundation for the results and conclusions they present. This is a very welcome and solid contribution to the multiple co-benefits evident from those practicing regenerative agriculture as it is conducted by the agricultural community.

Is the work clearly and accurately presented and does it cite the current literature?

Yes

If applicable, is the statistical analysis and its interpretation appropriate?

Yes

Are all the source data underlying the results available to ensure full reproducibility?

Yes

Is the study design appropriate and is the work technically sound?

Yes

Are the conclusions drawn adequately supported by the results?

Yes

Are sufficient details of methods and analysis provided to allow replication by others?

Yes

Reviewer Expertise:

I am a research scientist in the Texas A&M AgriLife Research organization working on documenting and testing causal mechanisms in comparing results from soil, vegetation, livestock, watershed health, and ecological economics. I have published widely on these aspects of regenerative agriculture with many co-authors for 5 decades.

I confirm that I have read this submission and believe that I have an appropriate level of expertise to confirm that it is of an acceptable scientific standard.

# Peer review: Reviewer response for version 1

**DOI:** 10.5256/f1000research.31489.r79606

**Published:** 2021-03-09

**Authors:** John M. Holland, Jenny Bussell

**Affiliations:** 1 Farmland Ecology Unit, Game and Wildlife Conservation Trust, Fordingbridge, UK; 2 The Allerton Project, Game and Wildlife Conservation Trust, Leicestershire, UK

A regenerative agriculture matrix scoring system is proposed that can be used to evaluate the extent to which a farming system has adopted regenerative practices. This is an important study as it defines a standard for regenerative systems in a similar way to those for other farming systems such as organic using simple scoring matrix that can be used by farmers. This has the advantage that they can monitor the progress of their system over time and adjust accordingly.

 This paper represents a substantial amount of work, covering a large number of sites, fully replicated studies with a lot of samples and appropriate experimental designs. It includes three food production systems. The analysis was relevant and is described in good detail, and the statistics employed are robust. The paper uses a novel approach to overcome the problems of comparing the overall outcomes from the regenerative agriculture system between farms, with many different variables. The authors focus on methods that underly the principles of regenerative agriculture rather than defining it as a set of practices. This was achieved by defining a matrix scoring system compiled from sums of regenerative and conventional practices and looking for correlations between the matrix scores with detailed biological and physical measurements. This enabled them to compare a range of systems that exhibit few or many of the regenerative agriculture practices, and regress these with a large number of soil health; plant, insect and microbial diversity; pests and profitability. Much useful information is provided on the different methodologies and their use in farming systems research.

 There are some limitations to the approach, especially with respect to farms that may be in transition to regenerative agriculture and are achieving this through slowly reducing reliance on agrochemicals. Therefore, with such farms their matrix scores may remain high despite achieving substantial decreases in agrochemicals because 4 of the 9 points depending on not using inorganic fertilisers, herbicides, fungicides and insecticides. Nevertheless, the paper provides a contribution to scientific knowledge by allowing some broad comparisons to be made on the overall benefits of the regenerative agriculture approach.

Is the work clearly and accurately presented and does it cite the current literature?

Yes

If applicable, is the statistical analysis and its interpretation appropriate?

Yes

Are all the source data underlying the results available to ensure full reproducibility?

Yes

Is the study design appropriate and is the work technically sound?

Yes

Are the conclusions drawn adequately supported by the results?

Yes

Are sufficient details of methods and analysis provided to allow replication by others?

Yes

Reviewer Expertise:

Agroecologist, entomologist, soil scientist

We confirm that we have read this submission and believe that we have an appropriate level of expertise to confirm that it is of an acceptable scientific standard.
